# Prognostic Value of Combined Analysis of CTLA-4 and PLR in Esophageal Squamous Cell Carcinoma (ESCC) Patients

**DOI:** 10.1155/2019/1601072

**Published:** 2019-08-14

**Authors:** Cui-Ying Zhang, Juan Zhang, Yun-Fan Ma, Hong Zhe, Ren Zhao, Yan-Yang Wang

**Affiliations:** ^1^Department of Radiation Oncology, General Hospital of Ningxia Medical University, Yinchuan, 750004 Ningxia, China; ^2^Department of Radiation Oncology, General Hospital of Chinese Armed Police Force Border Defence Force, Shenzhen, 518000 Guangdong, China; ^3^Graduate School, Ningxia Medical University, Yinchuan, 750004 Ningxia, China; ^4^Department of Thoracic Surgery, General Hospital of Ningxia Medical University, Yinchuan, 750004 Ningxia, China

## Abstract

**Objective:**

The purpose of this study was to evaluate the prognostic role of the cytotoxic T-lymphocyte-associated antigen-4 (CTLA-4) expression level and the platelet lymphocyte ratio (PLR) level in esophageal squamous cell carcinoma (ESCC) patients.

**Methods:**

84 ESCC patients who received surgical treatment in our hospital were enrolled in the study. The correlation of each biomarker's level with ESCC patients' clinicopathological characteristics and overall survival (OS) was assessed.

**Results:**

The elevated expression rate of T-CTLA-4 (tumor cell CTLA-4) and I-CTLA-4 (interstitial lymphocyte CTLA-4) was 48.8% and 44.0%, respectively. The number of enrolled patients with a higher PLR level (≥119) was 48. The prognostic value of T-CTLA-4, I-CTLA-4, and PLR in ESCC patients was not detected. However, patients with both a low T-CTLA-4 expression level and a low PLR level that had longer OS (*p* = 0.023) were found. The prognostic role of T-CTLA-4(-) +PLR (-) status in ESCC patients was also confirmed in multivariate analyses (*p* = 0.027).

**Conclusion:**

These results demonstrated the potential prognostic value of combined analysis of CTLA-4 and PLR in ESCC patients.

## 1. Introduction

Esophageal squamous cell carcinoma (ESCC) is one of the most common types of cancer in northern China [1, 2]. Although much progress has been made in surgical techniques and chemoradiotherapy, the survival rate of patients with ESCC is still low [3–5]. Therefore, developing novel prognostic factors and optimizing the treatment of ESCC patients are urgently needed [6].

Cytotoxic T-lymphocyte-associated antigen-4 (CTLA-4) is a critical immune checkpoint that negatively regulates T cell activation of the immune system. It has been reported that CTLA-4 is overexpressed and correlated with poor prognosis in various types of cancer, including breast cancer [7], lung cancer [8], prostate cancer [9], and cervical cancer [10]. More recently, promising results were also shown by administration of CTLA-4 inhibitor in cancer treatment [11–13]. Hence, the expression level of CTLA-4 in cancer cells or microenvironments may constitute a potential predictive or prognostic biomarker of malignant disease patients. However, the potential role of CTLA-4 in ESCC patients has yet to be clearly defined [14].

Previous studies have shown that systemic inflammatory factors can be used as a predictive or prognostic marker of immunotherapy patients targeting the CTLA-4 pathway [15–18]. These results suggest that there may be an interaction between systemic inflammatory factors and immune checkpoints. Therefore, the combined analysis of systemic inflammatory factors and immune checkpoint molecules may provide a new perspective for the study of prognostic biomarkers in cancer patients. As a systemic inflammatory factor, the prognostic significance of platelet lymphocyte ratio (PLR) in patients with ESCC has been evaluated in a number of retrospective studies [19–21]. However, the potential relationship between CTLA-4 and PLR in ESCC patients and its prognostic significance have not been revealed.

In this study, the prognostic value of the CTLA-4 expression level and the PLR level was analyzed in ESCC patients. The results showed that neither CTLA-4 nor PLR could predict the treatment outcome of ESCC patients. However, patients with both a low T-CTLA-4 expression level and a low PLR level that had longer overall survival (OS) were found in this study.

## 2. Materials and Methods

### 2.1. Patient's Characteristics

We retrospectively analyzed 84 patients with ESCC who underwent radical esophagectomy at the General Hospital of the Ningxia Medical University from September 2008 to December 2010. All of the enrolled patients had complete medical records and sufficient paraffin-embedded tissue blocks. Clinical, laboratory, pathological, and treatment data were collected from the patients' medical records. There were 74 males and 10 females with a median age of 63 years (range, 38–80 years). Tumor staging was classified according to the seventh edition of the American Joint Committee on Cancer Staging Manual [22]. There were 23 cases of stage I, 31 cases of stage II, and 30 cases of stage III. Tumor differentiation was classified as well and moderately (*n* = 50) or poorly differentiated (*n* = 34). Ivor Lewis esophagectomy, including two-field lymphadenectomy with total mediastinal lymph node dissection, was performed. Patients with postoperative pathological diagnosis of stage T3 or lymph node metastasis received adjuvant radiotherapy or chemotherapy. The percentage of patients receiving adjuvant radiotherapy and chemotherapy was 29.8% and 27.4%, respectively. All the enrolled patients did not receive radiotherapy or chemotherapy before surgery. The study protocol was approved by the Ethics Committee of the General Hospital of Ningxia Medical University.

### 2.2. Immunohistochemistry Staining and Analysis

Immunohistochemistry (IHC) was performed as previously reported [23]. Briefly, antigen retrieval was carried out in EDTA solution at 100°C, pH 9.0 for 10 minutes. After blocking of the nonspecific antigens, the tissue section was incubated with CTLA-4 antibody (Abcam, Cambridge, UK) overnight at 4°C. For antibody visualization, liquid 3,30-diaminbenzidine (DAKO) was used.

Tumor cells and interstitial lymphocytes were stained with CTLA-4. The expression levels of CTLA-4 were defined as follows: “−” (negative), “+” (weakly positive), “++” (moderately positive), and “+++” (strongly positive). Tissue sections with “++” and “+++” were defined as a high expression of CTLA-4 [24]. The IHC analysis was performed by two independent pathologists blinded to the clinical information.

### 2.3. PLR Level Evaluation

PLR is defined as the ratio of absolute platelet count to lymphocyte count. In each patient, the whole blood cells were detected within one week before the surgery and the platelet and lymphocyte counts were obtained. The cut-off value of PLR was determined by receiver operating curve (ROC) analysis.

### 2.4. Patient Follow-Up

Follow-up began from the date of completed surgery with or without chemoradiotherapy and continued until the last follow-up date or death of the patient. Follow-ups were conducted every three months in the first year, every six months in the next two years, and annually thereafter. OS is defined as the interval between the diagnosis of ESCC and the date of death or censored. The median follow-up time was 79 months.

### 2.5. Statistical Analysis

Pearson's chi-squared test or Fisher's exact test was used to analyze the relationship between CTLA-4 or PLR and clinicopathological features of ESCC patients. Spearman's rank correlation analysis was used to analyze the correlation between T-CTLA-4 (tumor cell CTLA-4), I-CTLA-4 (interstitial lymphocyte CTLA-4), and PLR. The OS curve was drawn by the Kaplan-Meier method and compared between groups by the log-rank test. The median follow-up time was also analyzed by the Kaplan-Meier method. The Cox proportional hazard model was used for univariate and multivariate analyses. The minimum significant level was described as *p* < 0.05. SPSS 22.0 (SPSS, Chicago, IL, USA) and GraphPad Prism 5.0 (La Jolla, CA, USA) were used for statistical analysis.

## 3. Results

### 3.1. CTLA-4 Expression Level and PLR Level in ESCC Patients

**Figure 1:**
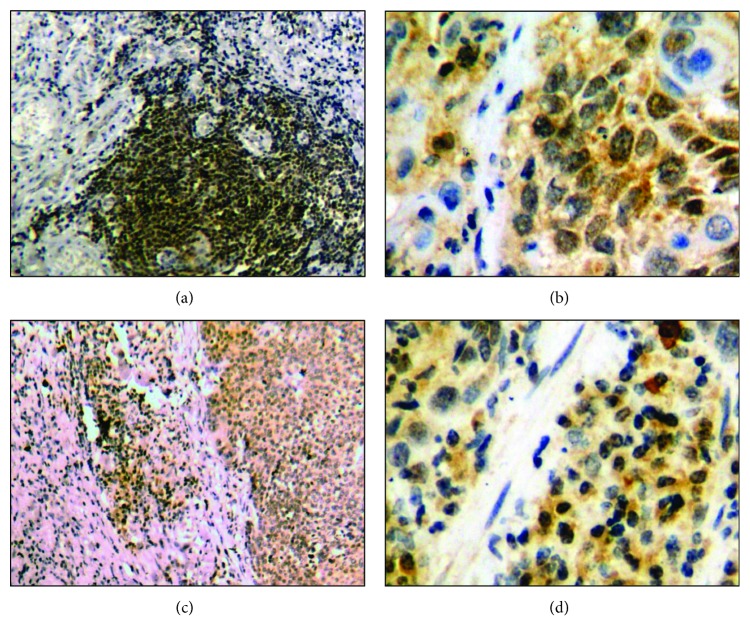
Representative immunostaining for T-CTLA-4 and I-CTLA-4. Positive staining for CTLA-4 in tumor cells ((a) 200x magnification; (b) 400x magnification). Positive staining for CTLA-4 in interstitial lymphocytes ((c) 200x magnification; (d) 400x magnification).

T-CTLA-4 and I-CTLA-4 were mainly distributed in the cytoplasm. The cytoplasmic pattern of I-CTLA-4 was more homogeneous than that of T-CTLA-4 staining.  Figure 1 shows the typical staining patterns for T-CTLA-4 and I-CTLA-4. The percentage of patients with high T-CTLA-4 and I-CTLA-4 expression was 48.8% and 44.0%, respectively.

**Figure 2:**
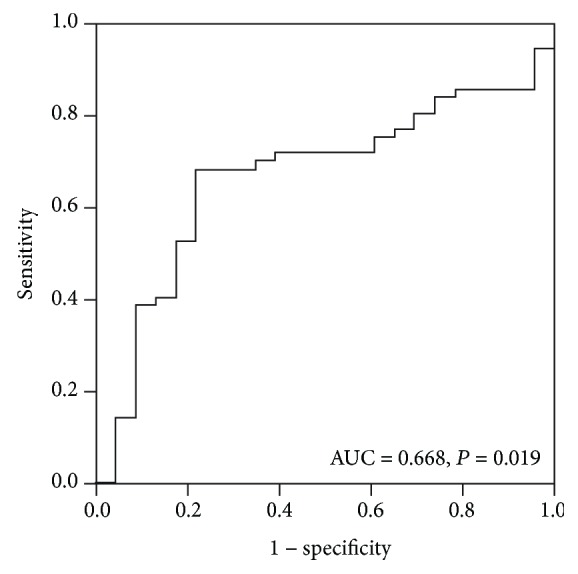
ROC curve. The optimal cut-off value of PLR was determined by ROC analysis.

The median PLR value of the whole group was 121.7. According to the results of ROC analysis (Figure 2), the cut-off value of PLR was determined to be 119. The number of patients with higher PLR levels was 48.The correlation between PLR and the expression of T-CTLA-4 or I-CTLA-4 was analyzed by Spearman's rank correlation. There was a positive correlation between the PLR level and the I-CTLA-4 expression level (*p* = 0.001). No correlation was found between PLR and T-CTLA-4 expression (*p* = 0.238).

### 3.2. The Impact of the CTLA-4 and PLR Level on the Survival of ESCC Patients

**Figure 3:**
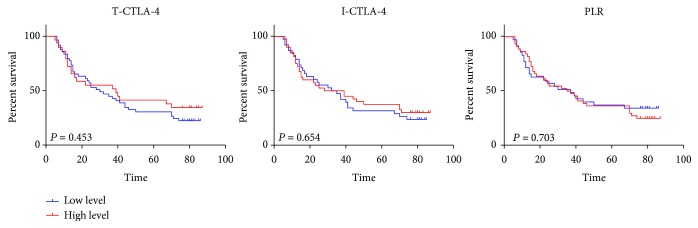
The overall survival curve of ESCC patients with different levels of T-CTLA-4, I-CTLA-4, and PLR. The Kaplan-Meier survival curve for patients with ESCC whose tumors were classified as either higher or lower level for T-CTLA-4, I-CTLA-4, and PLR, respectively. T-CTLA-4 (*p* = 0.453), I-CTLA-4 (*p* = 0.654), and PLR (*p* = 0.703) status did not demonstrate a significant relation with patient survival.

The effects of the CTLA-4 expression level and the PLR level on the survival of ESCC patients were examined. Kaplan-Meier analysis did not find the effects of the T-CTLA-4 expression level (*p* = 0.453), I-CTLA-4 expression level (*p* = 0.654), and PLR level (*p* = 0.703) on the OS in ESCC patients (Figure 3).

### 3.3. The Combined Analysis of CTLA-4 and PLR in ESCC Patients without Concurrent Inflammatory Disease

**Figure 4:**
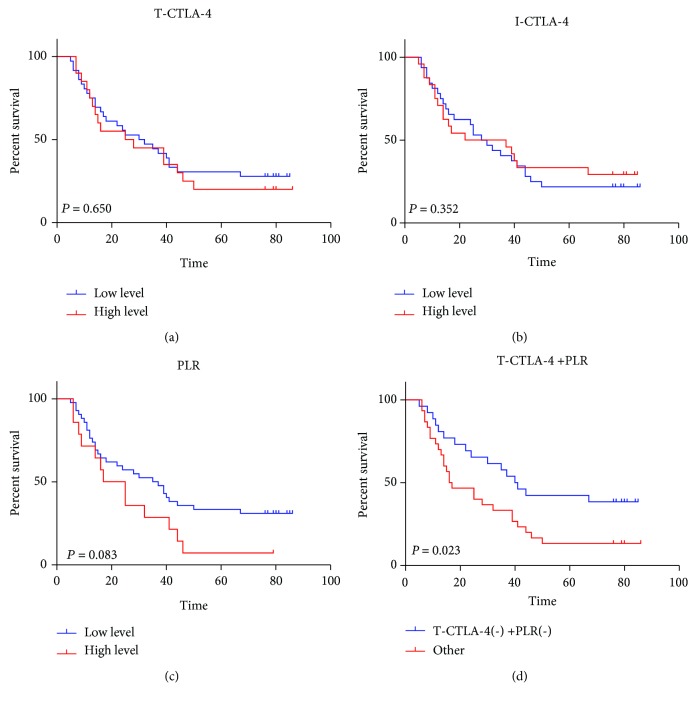
Patients with T-CTLA-4(-) +PLR (-) status had superior overall survival. Effects of different levels of T-CTLA-4 (a), I-CTLA-4 (b), PLR (c), and T-CTLA-4(-) +PLR (-) status (d) on the overall survival of ESCC patients without inflammatory diseases. Patients with T-CTLA-4(-) +PLR (-) status had significantly longer overall survival (*p* = 0.023).

Considering the interaction between inflammatory diseases and CTLA-4 or PLR levels, 12 ESCC patients with concurrent inflammatory diseases were excluded in the subsequent analysis. The expression levels of T-CTLA-4 (*p* = 0.650) (Figure 4(a)), I-CTLA-4 (*p* = 0.352) (Figure 4(b)), and PLR (*p* = 0.083) (Figure 4(c)) still had no effect on the OS in patients with ESCC without the concurrent inflammatory diseases. However, patients with both low T-CTLA-4 levels and low PLR levels (T-CTLA-4 (-) +PLR (-)) had superior OS (*p* < 0.023) than other patients (Figure 4(d)).

Univariate analysis showed that T stage (*p* = 0.012), N stage (*p* < 0.001), TNM stage (*p* < 0.001), adjuvant radiotherapy (*p* = 0.022), and T-CTLA-4(-) +PLR (-) status (*p* = 0.027) were significantly associated with OS. The multivariate analysis results showed that adjuvant radiotherapy (*p* = 0.023) and T-CTLA-4(-) +PLR (-) status (*p* = 0.030) were two independent prognostic factors (Table 1). However, no correlation between T-CTLA-4(-) +PLR (-) status and clinical characteristics were found in Pearson's chi-squared test or Fisher's exact test (Table 2).

## 4. Discussion

The CTLA-4 blockade has become a progressive treatment strategy and has opened up an exciting way for cancer management, including esophageal cancer [25]. The combination of immunotherapy and traditional treatment strategy can improve the therapeutic effect and prolong the life of patients with esophageal cancer. The analysis of the expression level of CTLA-4 in tumor cells and interstitial lymphocytes can provide a basis for the selection of patients with the most effective immunotherapy [26]. In addition, the prognostic value of the CTLA-4 expression in patients with ESCC should also be confirmed.

In this study, we first evaluated the prognostic value of CTLA-4 in patients with ESCC. The elevated expression rate of T-CTLA-4 and I-CTLA-4 was 48.8% and 44.0%, respectively. The prognostic value of T-CTLA-4 and I-CTLA-4 in ESCC was not detected. In considering the interaction between inflammation and the CTLA-4 expression, we excluded 12 ESCC patients with concurrent inflammatory diseases. However, T-CTLA-4 and I-CTLA-4 still had no effect on the outcome of the rest of the patients. In addition, no correlation was found between the expression of CTLA-4 and the clinical characteristics of patients with ESCC. So far, there is only one report about the expression of CTLA-4 in esophageal cancer, in which the expression level of CTLA-4 protein in primary esophageal cancer lesions has a potential prognostic value [14]. However, the relationship between the high expression of CTLA-4 and clinicopathological factors has not been reported. The difference between the published study and our study may be due to heterogeneous study samples, technical problems, and different cut-off values of the CTLA-4 expression. Therefore, the prognostic value of CTLA-4 in patients with ESCC remains to be evaluated in further studies.

Inflammation plays a key role in the development of cancer [27]. Chronic inflammation induced by environmental exposure can activate the proinflammatory signaling pathway, thereby promoting tumor growth, progression, angiogenesis, and metastasis [28, 29]. As an indicator of systemic inflammation, PLR has been widely studied in patients with ESCC, and its prognostic effect has been documented [19–21]. However, the results of different studies are controversial. In the current study, we have not revealed the prognostic value of PLR in patients with ESCC. In a recent meta-analysis, the authors suggest that a combined analysis of PLR and other biomarkers may play a role in distinguishing ESCC patients with different treatment outcomes [30].

Immune status has a great impact on the prognosis of cancer patients [31, 32]. The immune effect of the host and the cancer itself is two important aspects of the immune status that affect the cancer prognosis, and there is an interaction between them [33]. Understanding the interaction between the cancer and the host immune system holds a great promise to develop the biomarkers for the diagnosis, treatment, and prognosis evaluation of cancer patients [34]. In fact, studies have shown that there is an interaction between PLR, which reflects the host immune and inflammatory levels, and CTLA-4, which reflects the immune status of cancer [35–38]. Combined analysis of PLR and CTLA-4 can improve the effectiveness of prognostic evaluation in patients with ESCC. Therefore, we analyzed the CTLA-4 expression levels and the PLR levels in patients with ESCC in this study. From this analysis, we identified a subgroup of patients with a lower T-CTLA-4 level and a lower PLR level as having the best OS compared with other patients. Multivariate survival analysis also showed the effect of T-CTLA-4(-) +PLR (-) status on prognosis.

## 5. Conclusion

To the best of our knowledge, this is the first study to analyze the effect of CTLA-4 and PLR on the prognosis of patients with ESCC. However, further prospective studies are needed to confirm the results of this study and to explore whether it can guide personalized immunotherapy in patients with ESCC.

## Figures and Tables

**Table 1:** Univariate and multivariate Cox regression analyses estimating the risk factors of OS in ESCC patients.

Variable	Univariate analyses			Multivariate analyses		
	HR	95% CI	*p* value	HR	95% CI	*p* value
Gender	0.389	0.094-1.612	0.193			
Age	0.683	0.268-1.738	0.424			
KPS	1.237	0.674-2.271	0.493			
Drinking	0.943	0.505-1.760	0.854			
Smoking	1.449	0.762-2.756	0.258			
Weight loss	1.116	0.579-2.152	0.742			
Histological grade	0.832	0.454-1.527	0.553			
Location	2.706	0.652-11.237	0.171			
Length	1.991	0.949-4.177	0.069			
T stage	2.718	1.250-5.913	0.012	3.168	0.320-31.376	0.324
N stage	3.464	1.841-6.517	0.000	5.194	0.547-49.265	0.151
TNM stage	2.218	1.428-3.171	0.000	0.537	0.064-4.508	0.567
Resection margin	0.935	0.226-3.875	0.926			
Adjuvant radiotherapy	2.107	1.116-3.978	0.022	2.104	1.109-3.993	0.023
Adjuvant chemotherapy	1.829	0.941-3.556	0.075			
T-CTLA-4(-) +PLR (-)	2.031	1.083-3.807	0.027	2.025	1.071-3.826	0.030

Abbreviations: OS: overall survival; ESCC: esophageal squamous cell carcinoma; HR: hazard ratio; CI: confidence interval; KPS: Karnofsky; T-CTLA-4: tumor cell cytotoxic T-lymphocyte-associated antigen-4; PLR: platelet lymphocyte ratio.

**Table 2:** Clinicopathological characteristics of ESCC patients.

Clinicopathological characteristics	*N*	T-CTLA-4(-) +PLR (-)	Other	*p* value
Gender				
Male	66	30	36	
Female	6	2	4	0.686
Age (years)				
<70	62	27	35	
≥70	10	5	5	0.703
KPS				
>80%	39	17	22	
≤80%	33	15	18	0.874
Weight loss (kg)				
<5	50	22	28	
≥5	22	10	12	0.909
Drinking				
Yes	32	13	19	
No	40	19	21	0.560
Smoking				
Yes	46	21	25	
No	26	11	15	0.784
Histological differentiation				
Well and moderate	39	15	24	
Poor	33	17	16	0.267
Location				
Proximal third	7	3	4	
Middle third	43	17	26	
Distal third	22	12	10	0.513
Tumor length (cm)				
<3	18	10	8	
≥3	54	22	32	0.273
Infiltration depth				
T1 and T2	21	11	10	
T3 and T4	51	21	30	0.384
Lymph node status				
N0	47	23	24	
N1	25	9	16	0.293
pTNM staging				
I and II	48	23	25	
III	24	9	15	0.402
Resection margin				
Positive	3	1	2	
Negative	69	31	38	1.000
Adjuvant radiotherapy				
Yes	19	7	12	
No	53	25	28	0.437
Adjuvant chemotherapy				
Yes	16	5	11	
No	56	27	29	0.228

Abbreviations: T-CTLA-4: tumor cell cytotoxic T-lymphocyte-associated antigen-4; PLR: platelet lymphocyte ratio; ESCC: esophageal squamous cell carcinoma; KPS: Karnofsky.

## Data Availability

The data used to support the findings of this study are available from the corresponding authors upon request.

## Supplementary Materials

Supplementary Materials Table S1: comparison of clinicopathologic characteristics between T-CTLA-4(-) +PLR (-) and other group of ESCC patients without concurrent inflammatory disease.
