# Supplementary material for: Prognostic Value of Combined Analysis of CTLA-4 and PLR in Esophageal Squamous Cell Carcinoma (ESCC) Patients
Source: Dis Markers. 2019 Aug 14;2019:1601072. doi: 10.1155/2019/1601072 (PMC6710793; doi:10.1155/2019/1601072)
Supplement: Supplementary Materials — Table S1: comparison of clinicopathologic characteristics between T-CTLA-4(-) +PLR (-) and other group of ESCC patients without concurrent inflammatory disease. [file 1601072.f1.pdf]

Table S1 Comparison of clinicopathologic characteristics between T-CTLA-4(-) +PLR (-) and other group of ESCC patients without concurrent inflammatory disease

Clinicopathologic characteristics	N	T-CTLA-4(-) +PLR (-)	Other	P value
Gender				
Male	54	25	29	
Female	4	1	3	0.620
Age (years)				
<70	50	22	28	
≥70	8	4	4	1.000
KPS				
>80%	31	14	17	
≤80%	27	12	15	0.956
Weight loss (kg)				
<5	40	18	22	
≥5	18	8	10	0.969
Drinking				
Yes	23	9	14	
No	35	17	18	0.479
Smoking				
Yes	37	17	20	
No	21	9	12	0.820
Histological differentiation				
Well and moderate	31	12	19	
Poor	27	14	13	0.315
Tumor length (cm)				
<3	14	8	6	
≥3	44	18	26	0.287
Infiltration depth				
T1 and T2	17	9	8	
T3 and T4	41	17	24	0.424
Lymph node status				
N0	38	19	19	
N1	20	7	13	0.275
pTNM staging				
I and II	39	19	20	0.393
III	19	7	12	

Abbreviations: T-CTLA-4, tumor cells cytotoxic T-lymphocyte-associated antigen-4; PLR, platelet lymphocyte ratio; ESCC, esophageal squamous cell carcinoma; KPS, Karnofsky
